# Polyunsaturated Fatty Acids Attenuate Diet Induced Obesity and Insulin Resistance, Modulating Mitochondrial Respiratory Uncoupling in Rat Skeletal Muscle

**DOI:** 10.1371/journal.pone.0149033

**Published:** 2016-02-22

**Authors:** Gina Cavaliere, Giovanna Trinchese, Paolo Bergamo, Chiara De Filippo, Giuseppina Mattace Raso, Giorgio Gifuni, Rosalba Putti, Bottu Heleena Moni, Roberto Berni Canani, Rosaria Meli, Maria Pina Mollica

**Affiliations:** 1 Department of Biology, University of Naples “Federico II”, Napoli, Italy; 2 Institute of Food Sciences, CNR-ISA, Avellino, Italy; 3 Department of Pharmacy, University of Naples “Federico II”, Napoli, Italy; 4 Department of Translational Medical Sciences, European Laboratory for Food Induced Diseases, University of Naples “Federico II”, Napoli, Italy; Monash University, AUSTRALIA

## Abstract

**Objectives:**

Omega (ω)-3 polyunsaturated fatty acids (PUFA) are dietary compounds able to attenuate insulin resistance. Anyway, the precise actions of ω-3PUFAs in skeletal muscle are overlooked. We hypothesized that PUFAs, modulating mitochondrial function and efficiency, would ameliorate pro-inflammatory and pro-oxidant signs of nutritionally induced obesity.

**Study Design:**

To this aim, rats were fed a control diet (CD) or isocaloric high fat diets containing either ω-3 PUFA (FD) or lard (LD) for 6 weeks.

**Results:**

FD rats showed lower weight, lipid gain and energy efficiency compared to LD-fed animals, showing higher energy expenditure and O_2_ consumption/CO_2_ production. Serum lipid profile and pro-inflammatory parameters in FD-fed animals were reduced compared to LD. Accordingly, FD rats exhibited a higher glucose tolerance revealed by an improved glucose and insulin tolerance tests compared to LD, accompanied by a restoration of insulin signalling in skeletal muscle. PUFAs increased lipid oxidation and reduced energy efficiency in subsarcolemmal mitochondria, and increase AMPK activation, reducing both endoplasmic reticulum and oxidative stress. Increased mitochondrial respiration was related to an increased mitochondriogenesis in FD skeletal muscle, as shown by the increase in PGC1-α and -β.

**Conclusions:**

our data strengthened the association of high dietary ω3-PUFA intake with reduced mitochondrial energy efficiency in the skeletal muscle.

## Introduction

When food intake chronically exceeds metabolic needs, efficient metabolism causes continued energy storage and results in obesity, a common condition associated with diabetes, hyperlipidemia and inflammatory state.

Although the peripheral insulin resistance is still not fully understood, several mechanisms have been proposed, including an increase of intracellular lipid metabolites, inflammation, endoplasmic reticulum (ER) stress and mitochondrial dysfunction [1–3]. In particular, ER stress appears to act directly as a negative modulator of insulin signalling, and indirectly promoting lipid accumulation [4,5]. Activation of AMP-activated protein kinase (AMPK) protects against lipid-induced hepatic [6] and skeletal muscle disorders, reducing ER stress [7].

The beneficial effects of adiponectin, including anti-inflammatory and insulin-sensitizing actions, have been well established [8–10]. Adiponectin plays an important role in the pathophysiology of diabetes in obesity, at least in part via regulating metabolism in skeletal muscle [9]. Adiponectin, as well as leptin, directly modulate fatty acid metabolism in skeletal muscle by increasing fatty acid oxidation and suppressing fatty acid synthesis via mechanisms involving AMPK activation [11,12]. Adiponectin also confers beneficial metabolic effects in muscle by enhancing mitochondrial biogenesis [13].

Mitochondria are at the centre of glucose and fatty acid metabolism. In fact, mitochondrial dysfunction, increased production of reactive oxygen species (ROS), and impaired mitochondrial biogenesis are considered the key determinants of insulin resistance and obesity [2,3].

Mitochondrial uncoupling, which reduces the proton gradient across the mitochondrial inner membrane, creates a futile cycle of glucose and fatty acid oxidation without generating ATP [14–17], increasing lipid oxidation and reducing intracellular lipid content [18,19]. Promoting inefficient metabolism, such as the generation of heat instead of ATP, is a potential treatment for obesity. In fact, the modulation of mitochondrial function and efficiency has been suggested for the prevention/treatment of obesity and insulin resistance [20]; therefore, drugs or natural molecules modulating the mitochondrial function and efficiency may be useful in the treatment/prevention of obesity and insulin resistance.

The ω-3 polyunsaturated fatty acids (PUFA), docosahexaenoic acid (DHA) and eicosapentaenoic acid (EPA), are dietary compounds that are intensively studied as potent anti-inflammatory products, able to reduce the risk of insulin resistance and ameliorate obesity-associated disorders through affecting hormonal control and modulating AMPK activity [21, 22].

Recently, we have demonstrated that the replacement of lard, rich in saturated fatty acids (SFA), with fish oil (rich in ω-3 PUFA) in high-fat diet is able to limit the development of systemic and tissue inflammation [23]. In addition, the reduction of hepatic lipid accumulation by PUFAs resulted by an improved mitochondrial fatty acid utilization associated with mitochondrial mild uncoupling, which counteracted the hepatocyte damage induced by long-term over-feeding [24].

Skeletal muscle, the main site of insulin-mediated glucose disposal and triglyceride clearance, is another attractive site for altering metabolism and adiposity, through engineered respiratory uncoupling. In fact, skeletal muscle is a chief determinant of resting metabolic rate, whose reduction is associated with weight gain [25].

Here, we hypothesized that the ω-3 PUFAs, showing an increased ability to modulate mitochondrial function and efficiency, would also ameliorate pro-inflammatory and pro-oxidant signs of fat overnutrition. To test this hypothesis, rats were fed a control diet (CD) or high fat isocaloric diets containing either ω-3 PUFA (FD) or lard (LD) for 6 weeks. We focused on mitochondrial function, efficiency and biogenesis of mitochondria located beneath the sarcolemmal membrane (subsarcolemmal [SS]) or between the myofibrils (intermyofibrillar [IMF]) in skeletal muscle. In fact, these two mitochondrial populations exhibit different energetic characteristics [26,27] and therefore can be differently affected by physio-pathological stimuli. Finally, cytoprotective enzymes activities and ER stress modulation by ω-3 PUFAs were analyzed.

## Materials and Methods

### Ethics statement

Procedures involving animals and their care were conducted in conformity with international and national law and policies (EU Directive 2010/63/EU for animal experiments, ARRIVE guidelines and the Basel declaration including the 3R concept). The procedures reported here were approved by the Institutional Committee on the Ethics of Animal Experiments (CSV) of the University of Naples “Federico II” and by the Ministero della Salute.

### Experimental procedures

All chemicals were purchased by Sigma–Aldrich (St. Louis, MO, USA), unless otherwise specified. Young male Wistar rats (60 days old; 345 ± 7 g; Charles River, Calco, Lecco, Italy) were individually caged in a temperature-controlled room and exposed to a daily 12h–12h light–dark cycle with free access to chow diet and drinking water. Rats were divided into 3 experimental groups (n = 8) according to a different 6 weeks dietary regimen: the first group (control diet, CD) received a standard diet (10.6%fat J/J); the second group (LD) received the high fat diet rich in lard (40% fat J/J); the third group (FD) received the high fat diet rich in fish oil (40% fat J/J). The composition of all dietary regimens is reported in Tables 1–2. An additional group (n = 8) was sacrificed at the beginning of the study to establish baseline measurements of body compositions.

**Table 1:** Diet Composition.

	Control Diet	High Lard Diet (g/100g diet)	High Fish oil Diet (g/100g diet)
**Standard feed**	100	51,3	51,3
**Casein^a^ g**	-	9,25	9,25
**Lard g**	-	21,8	-
**Fish oil ^b^ g**	-	-	21,8
**Sunflower oil g**	-	1,24	1,24
**AIN 76 mineral mix^c^ g**	-	1,46	1,46
**AIN 76 Vitamin mix^d^ g**	-	0,42	0,42
**Choline bitartrate**	-	0,08	0,08
**Methionine g**	-	0,12	0,12
**Energy density kJ/g diet**	15,88	20,00	20,00
**Protein %**	29	29	29
**Lipid %**	10,6	40	40
**Carbohydrate %**	60,4	31	31

^a^Purified high-nitrogen casein containing 88% protein

^b^Fish oil = Cod liver Oil

^c^American Institute of Nutrition (1977)

^d^American Institute of Nutrition (1980).

**Table 2:** Fatty acid composition (g/100g fatty acid) of experimental diets.

Fatty acid	High lard diet	High fish oil diet
**SFA**	42,64	17,87
**10:00**	0,05	0,00
**12:00**	0,11	0,00
**14:00**	0,76	2,12
**16:00**	26,89	9,62
**18:00**	14,83	2,8
**MUFA**	34,18	35,16
**16:1**	1,53	4,94
**18:1**	31,87	19,63
**20:1**	0,66	6,24
**22:1**	0,00	4,35
**PUFA**	22,94	47,14
**18:2**	20,57	17,1
**18:3**	2,4	5,32
**18:4**	0,00	0,56
**20:4**	0,00	0,56
**20:5- n3**	0,00	8,1
**22:5- n3**	0,00	1,2
**22:6- n3**	0,00	14,3

MUFA, monounsaturated fatty acids; PUFA, polyunsaturated fatty acids; SFA, saturated fatty acids

After 6 weeks feeding, the animals were anaesthetised by injection of chloral hydrate (40 mg/100 g body weight, i.p.), and blood was taken from both the inferior cava and portal vein. Skeletal muscle was removed; samples not immediately used for mitochondrial preparation were frozen and stored at -80°C.

### Evaluation of body composition and energy balance

During the experimental time, the body weight and food intake were monitored daily to calculate weight gain and gross energy intake. Spilled food and faeces were collected daily for precise food intake calculation. Energy balance assessments were conducted over the 6 weeks of feeding by the comparative carcass evaluation [28]. The gross energy density for the standard diet or high fat diets (15.8 or 20.0 kJ/g, respectively), as well as the energy density of the faeces and the carcasses, were determined by bomb calorimetric (Parr adiabatic calorimetric, Parr Instrument Co., Moline, IL, USA). Energy, fat and protein content in animal carcasses were measured, according to Iossa et al. [28]. Metabolizable energy (ME) intake was determined by subtracting the energy measured in faeces and urine from the gross energy intake, which was determined from the daily food consumption and gross energy density. Energy efficiency was calculated as the percentage of body energy retained per ME intake, and energy expenditure was determined as the difference between ME intake and energy gain.

### Measurement of oxygen consumption (VO_2_), carbon dioxide production (VCO_2_) and respiratory quotient (RQ)

Upon an adaption period to the experimental environment (at least 1 day), VO_2_ and VCO_2_, were recorded by a monitoring system (Panlab s.r.l., Cornella, Barcelona, Spain) composed of a four-chambered indirect open-circuit calorimeter, designed for continuous and simultaneous monitoring. VO_2_ and VCO_2_ were measured every 15 min (for 3 min) in each chamber for a total of 6 hours (from 8:00 am to 14:00 pm). The mean VO_2_, VCO_2_ and RQ values were calculated by the “Metabolism H” software [29].

### Oral glucose tolerance test, insulin tolerance test, and serum analysis

For the oral glucose tolerance test, overnight fasted rats received oral administration of glucose (3 g/kg body wt) dissolved in water. For the insulin tolerance test, rats were fasted for 5 h and then injected intraperitoneally with insulin (homolog rapid-acting, 10 units/kg body wt in sterile saline; Novartis, Basel, Switzerland). Samples of blood were collected before the oral glucose and insulin tolerance tests and at various times thereafter, and glucose and insulin values were determined by means of a glucose monitor (BRIO, Ascensia, NY), calibrated for use with rats and ELISA (Mercodia rat insulin; Mercodia, Uppsala, Sweden), respectively.

Basal fasting values of serum glucose and insulin were used to calculate Homoeostatic Model Assessment (HOMA) index as (Glucose (mg/dL) * Insulin (mU/L))/405 [30].

Plasma concentrations of triglycerides and cholesterol, and non-esterified fatty acids (NEFAs) were measured by colorimetric enzymatic method using commercial kits (SGM Italia, Italy and Randox Laboratories ltd., United Kingdom). Commercially available ELISA kits were used to determine adiponectin and leptin (B-Bridge International, Mountain View, CA), lipopolysaccharide (LPS) (Limus Amebocyte Lysate QCL-1000, Lonza Group Ltd), TNF-α and monocyte chemoattractant protein (MCP)-1 (Thermo Scientific, Rockford, IL).

### Analysis in skeletal muscle and mitochondrial parameters

Hind leg muscles were freed of excess fat and connective tissue, finely minced and washed in a medium containing 100mM KCl, 50mM TRIS, pH 7.5, 5mM MgCl2, 1mM EDTA, 5mM EGTA, 0.1% (w/v) fatty acid free bovine serum albumin (BSA). Tissue fragments were homogenized with the above medium (1: 8, w/v) in a Potter Elvehjem homogenizer (Heidolph, Kelheim, Germany) set at 500 rpm (4 strokes = min) and filtered through sterile gauze.

Homogenate was then centrifuged at 500×g for 10 min and the resulting precipitate was subsequently used for the preparation of the IMF mitochondria. The supernatant was centrifuged at 3000×g for 10 min and the resulting pellet, containing SS mitochondria, was washed twice and resuspended in suspension medium. The pellet from the 500×g centrifugation was resuspended in a small amount of homogenisation solution and treated with protease nagarse (9 U/g tissue) for 5 min. The suspension was then homogenised, filtered through sterile gauze and centrifuged at 3000×g for 10 min. The resulting supernatant was rapidly discarded and the pellet was resuspended and centrifuged at 500×g for 10 min. The supernatant containing the IMF mitochondria was centrifuged at 3000×g for 10 min, the pellet was washed once and resuspended in suspension medium. Mitochondrial oxygen consumption and basal/palmitate induced proton-leaks were evaluated [28]. Oxygen consumption was measured polarographically with a Clark-type electrode (Yellow Springs Instruments, Yellow Springs, Ohio) in a 3-ml glass cell, at a temperature of 30°C. Isolated SS or IMF mitochondria (0.1 mg protein/ml) were incubated in a medium containing 30 mM KCl, 6 mM MgCl_2_, 75 mM sucrose, 1 mM EDTA, 20 mM KH_2_PO_4_ pH 7.0, and 0.1% (w/v) fatty acid-free BSA. In the presence of 10 mM succinate, 3.75 mM rotenone and 0.6 mM ADP, state 3 oxygen consumption was measured. State 4 was obtained in the absence of ADP. The respiratory control ratio (RCR) was calculated as the ratio between states 3 and 4. In control experiments, we assured the quality of our mitochondrial preparation by checking that contamination of mitochondria by other ATPase-containing membranes was lower than 10%, and addition of cytochrome c (3 nmol/mg protein) only enhanced state 3 respiration by approximately 10%. Measurements of basal proton leak kinetics was performed as below reported. Mitochondrial oxygen consumption was measured polarographically, and membrane potential recordings were performed in parallel with safranin O using a Jasco dual-wavelength spectrophotometer (511–533 nm) (28). The absorbance readings were transferred to millivolt membrane potential using the Nernst equation,ΔΨ = 61 mV × log([K+]in/[K+]out), and calibration curves made for each preparation as previously reported (28). Measurements were carried out at 30°C in a medium containing 30 mmol/l LiCl, 6 mmol/l MgCl2, 75 mmol/l sucrose, 1 mmol/l EDTA, 20 mmol/l Tris-P, pH 7.0, and 0.1% (wt/vol) BSA in the presence of succinate (10 mmol/l), rotenone (3.75 μmol/l), oligomycin (2 μg/ml), safranin O (83.3 nmol/mg), and nigericin (80 ng/ml). Oxygen consumption and membrane potential were titrated by sequential additions of malonate up to 5 mmol/l for SS and 3 mmol/l for IMF mitochondria. Palmitate-induced proton leak kinetics was evaluated as above in the presence of palmitate (45 μmol/l and 65 μmol/l for SS and IMF mitochondria, respectively). Carnitine-palmitoyl-transferase (CPT) activity was followed spectrophotometrically as CoA-sH production by the use of 5,5'-dithiobis(nitrobenzoic acid) (DTNB) and as substrate palmitoyl coa 10 μM. The medium consisted of 50 mM KCl, 10 mM Hepes (pH 7.4), 0.025% Triton X-100, 0.3 mM DTNB, and 10–100 pg of mitochondrial protein in a final volume of 1.0 ml. The reaction was followed at 412 nm with spectrophotometer, and enzyme activity was calculated from an E412 = 13,600/ (M X cm). The temperature was thermostated to 25°C [31]. Determination of aconitase specific activity was carried out in a medium containing 30 mM sodium citrate, 0.6 mM MnCl2, 0.2 mM NADP, 50 mM TRIS-HCl pH 7.4, and 2 units of isocitrate dehydrogenase. The formation of NADPH was followed spectrophotometrically (340 nm) at 25°C. The level of aconitase activity measured equals active aconitase (basal level). Aconitase inhibited by ROS in vivo was reactivated so that total activity could be measured by incubating mitochondrial extracts in a medium containing 50 mM dithiothreitol, 0.2 mM Na2S, and 0.2 mM ferrous ammonium sulphate [32]. Rate of mitochondrial H_2_O_2_ release was assayed by following the linear increase in fluorescence (ex 312 nm and em 420 nm) due to the oxidation of homovanillic acid in the presence of horseradish peroxidase [33].

Skeletal muscle lipid content was determined using Folch method [34] and lipid droplets were assessed in haematoxylin-eosin stained sections. Adipocyte differentiation-related protein (ADRP) expression in rat gastrocnemius was assessed immunohistochemically and glycogen staining with periodic acid-Schiff (PAS).

Oxidative stress markers (Carbonylated Proteins, PC and the GSH/GSSG ratio) were measured in skeletal muscle. PC concentration was spectrophotometrically measured in blood plasma and tissue samples, and total thiols (GSH+GSSG) in plasma and the GSH and GSSG concentrations in rat muscles were determined with the dithionitrobenzoic acid (DTNB)-GSSG reductase recycling assay [35].

NF-E2-related factor 2 (Nrf2) is considered the main mediator of cellular adaptation to redox stress and its translocation into the nucleus, upon the dissociation from the Kelch-like ECH-associated protein 1 (Keap1), triggers the transcription of several enzymes involved in detoxification and chemopreventive mechanisms (glutathione S-transferases, GSTs; NAD(P)H:quinone oxidoreductase, NQO1; heme oxygenase-1). To investigate the possible involvement of NF-E2-related factor 2 (Nrf2) in the diet-induced stress, cytoplasmic and nuclear extracts were prepared from rat muscle tissue [35]. The enzymatic activities of GST and NADPH quinone oxidoreductase 1 (NQO1) were evaluated spectrophotometrically in cytoplasmic extracts [36–38] and the levels of Nrf2 levels in the nucleus were immunodetected by Western blotting analysis.

For p-Akt detection in skeletal muscle, additional six rats for each group were feed deprived for 6 h, then were administered insulin (10 U/Kg) and were killed 15 min after insulin injection for Western blot analysis.

### Western blot analysis

Skeletal muscle were homogenized and total protein lysates were subjected to SDS-PAGE. Blots were probed with p-Akt (Ser473) and Akt (Cell Signalling, MA, USA, diluted 1:1,000 in blocking buffer) or TNFα, BiP/ glucose regulated protein (GRP)78, p-eukaryotic translation initiation factor (eIF)2α, t-eIF2α, p-AMPK, t-AMPK (Santa Cruz Biotechnology, Santa Cruz, CA, USA). Western blot for tubulin or lamin was performed to ensure equal sample loading.

### Quantitative real-time PCR analysis

Total RNA was extracted from skeletal muscle using the TRIzol Reagent (Ambion). After DNase treatment (Ambion), RNA was quantified using a Nanodrop 2000c spectrophotometer (ThermoScientific) and reverse-transcribed (1 μg) using the Advantage RT-PCR kit (Clontech) and oligo dT primer.

Universal Probe Library Assay Design Center (https://www.roche-applied-cience.com/sis/rtpcr/upl/index.jsp?id=UP030000) was used for designing primers. The Real Time-PCR reactions were performed using a 7500 Real-Time PCR System (Applied Biosystems) in the presence of 1X Power Sybr Green PCR Master mix (Applied Biosystems) and 0.1 μM of each primer and 30 ng of cDNA. The thermal protocol was as follows: 2 min at 50°C, 10 min at 95°C, followed by 40 cycles of 15 sec at 95°C and 1 min at 60°C. For all of the genes examined, the reactions were conducted in technical duplicates. For each well, the evaluation of PCR efficiency and optimal threshold cycle (CT) of the target genes [peroxisome proliferator-activated receptor γ coactivator (PGC)1α, PGC1β, fibroblast growth factor (FGF)21] and the endogenous control gene (β-actin) were performed using the REAL TIME PCR MINER online tool [39]. The mean relative expression ratio (rER) of the target genes was calculated using β-actin as the endogenous control gene and cDNA as the reference sample applying the formula:

$\text{rER}\;=\;{\left(1\;+\;\text{E target gene}\right)}^{-\Delta \text{CT target gene}}/{\left(1\;+\;\text{E endogenous control}\right)}^{-\Delta \text{CT endogenous control}}$ [40], where ΔCT_target gene_ is the difference between the CT value of the target gene in the skeletal muscle of the FD, LD rats and the CT value of the target gene in the skeletal muscle of the control rats, ΔCT_endogenous control_ is the difference between the CT value of the β-actin gene in skeletal muscle of the FD, LD rats and the CT value of the β-actin gene in the skeletal muscle of the control rats [40]

Primer sequences used for Real-Time Polymerase Chain Reaction are the following:

**β-actin** 5’- ATTGCTGACAGGATGCAGAA-3’ and 5’- TAGAGCCACCAATCCACACAG-3’;

**FGF21** 5’- CACACCGCAGTCCAGAAAG-3’ and 5’- GGCTTTGACACCCAGGATT- 3’.

**PGC1-α** 5’- AAAGGGCCAAGCAGAGAGA-3’ and 5’- GTAAATCACACGGCGCTCTT-3’

**PGC1-β** 5’- TTGACAGTGGAGCTTTGTGG-3’ and 5’- GGGCTTATATGGAGGTGTGG-3’

### Statistical analysis

All data are presented as means±SEM. Differences among groups were compared by ANOVA followed by the Newman-Keuls test to correct for multiple comparisons. Differences were considered statistically significant at p<0.05. All analyses were performed using GraphPad Prism (GraphPad Software, San Diego, CA).

## Results

### Energy balance

As shown in Table 3, LD rats were characterised by a significant increase of body weight and weight gain, lipid accumulation and body energy levels compared to CD and FD animals; in addition, LD rats contained significantly lower percentages of water and protein compared to CD or FD rats. Despite a comparable ME intake, LD rats showed energy efficiency, body weight gain (expressed in g and kJ), lipid gain and lipid gain/ME intake values significantly higher than those of FD-fed animals. Decreased lipid accumulation in FD- compared to LD-fed animals was associated with higher energy expenditure, increased O_2_ consumption/CO_2_ production and lower energy efficiency. Moreover, LD and FD rats showed a lower respiratory quotient (RQ) compared to CD rats. Therefore, the low lipid gain and lipid gain/ME intake of FD animals (vs LD) indicates an improved ability to utilise fat, as a metabolic fuel.

**Table 3:** Body composition, energy balance, and calorimetric parameters.

	CD	LD	FD
**Body weight**			
**Initial body weight, g**	349±3,6^a^	343±4,5 ^a^	344±2,3 ^a^
**Final weight, g**	486±16.2 ^a^	636±14,0 ^b^	561±12,4 ^c^
**Body weight gain, g**	137,6±10.0 ^a^	293±12,0 ^b^	217±11 ^c^
**Body composition**			
**Water, %**	62.5±0.6^a^	54.8±2.2^b^	60.3±0.4^a^
**Lipid, %**	11.9±0.6^a^	22.2±1.6^b^	16.2±0.7^c^
**Protein, %**	18.4±0.2	15.7±0.9^b^	17.2±0.2^a^
**Body Energy kJ/g**	9.0±0.2	12.4±0.5^b^	10.3±0.3^c^
**Energy balance**			
**ME intake, kJ**	13442±403^a^	20333±508^b^	20306±211^b^
**Body weight gain, kJ**	1233±162^a^	3760±331^b^	2237±164^c^
**Energy efficiency %**	9.0±0.5^a^	18.0±1.1^b^	11.0±0.8^a^
**Protein gain kJ**	468±30^a^	377±30^b^	357±16^b^
**Lipid gain kJ**	864±134^a^	3483±422^b^	1873±162^c^
**Protein gain/ME intake %**	3.5±0.5^a^	1.9±0.5^b^	1.8±0.1^b^
**Lipid gain/ME intake %**	6.4±1.0^a^	17.1±1.7^b^	9.2±0.8^a^
**Energy expenditure kJ**	12209±450^a^	16572±280^b^	18063±224^c^
**Calorimetric parameters**			
**VO**_**2**_ **(ml/min/Kg**^**0.75**^**bw)**	6.6±0.2^a^	8.6±0.4^b^	11.8±0.9^c^
**VCO**_**2**_ **(ml/min/Kg**^**0.75**^**bw)**	6.0±0.3^a^	7.5±0.3^b^	10.3±0.4^c^
**RQ (VCO**_**2/**_**VO**_**2**_**)**	0.91±0.01^a^	0.87±0.01^b^	0.87±0.01^b^

ME = Metabolizable energy; Energy efficiency = ME intake/bw gain; VO_2_ = oxygen consumption; VCO_2_ = carbon dioxide production; RQ = respiratory quotient VCO_2_ /VO_2_.

Data are presented as means ± S.E.M. from n = 8 animals/group. Different superscripted letters indicate statistically significant differences (P < 0.05).

### Serum metabolite levels and glucose homeostasis

Blood parameters and hormonal determination are reported in Table 4. Serum metabolic (triglycerides, cholesterol, and insulin), and pro-inflammatory (TNFα, MCP1 and LPS) parameters in LD-fed animals were increased compared to those measured in rats on CD and, interestingly, no significant difference was shown in the same parameters between FD and CD. In contrast, NEFA and leptin concentrations were significantly higher after both high fat diet regimens compared to CD, however values from LD were significantly higher than those of FD rats. In addition, adiponectin concentration was reduced in LD compared to CD or FD animals, while glucose level was significantly increased both in LD or FD.

**Table 4:** Blood Parameters.

	CD	LD	FD
**NEFA (mmol/L)**	0.27±0.01^a^	0.43±0.02^b^	0.34±0.01^c^
**Triglycerides (mg/dL)**	51.6±4.0^a^	73.2±2.4^b^	55.0±3.0^a^
**Cholesterol (mg/dL)**	47.8±1.1^a^	72.2±8.7^b^	47.5±1.2^a^
**Leptin (ng/mL)**	10.7±0.9^a^	19.2±1.2^b^	14.1±1.2^c^
**Adiponectin (μg/mL)**	5.9±0.3^a^	4.3±0.6^b^	6.3±0.6^a^
**Glucose (mg/dL)**	84.1±2.6^a^	106.8±5.3^b^	105.8±7.3^b^
**Insulin (μg/L)**	0.6±0.2^a^	1.2±0.1^b^	0.6±0.1^a^
**TNFα (ng/ml)**	0.11±0.01^a^	0.21±0.02^b^	0.13±0.01^a^
**MCP1 (ng/mL)**	2.9±0.4^a^	7.4±0.8^b^	3.8±0.8^a^
**LPS (EU/mL)**	0.7±0.01^a^	0.9±0.01^b^	0.6±0.01^a^

NEFA: non-esterified fatty acids; TNF-α: tumor necrosis factor alpha; LPS: lipopolysaccharide; MCP-1: monocyte chemoattractant protein-1. Data are presented as means±S.E.M. from n = 8 animals/group. Different superscripted letters indicate statistically significant differences (P < 0.05).

**Fig 1:**
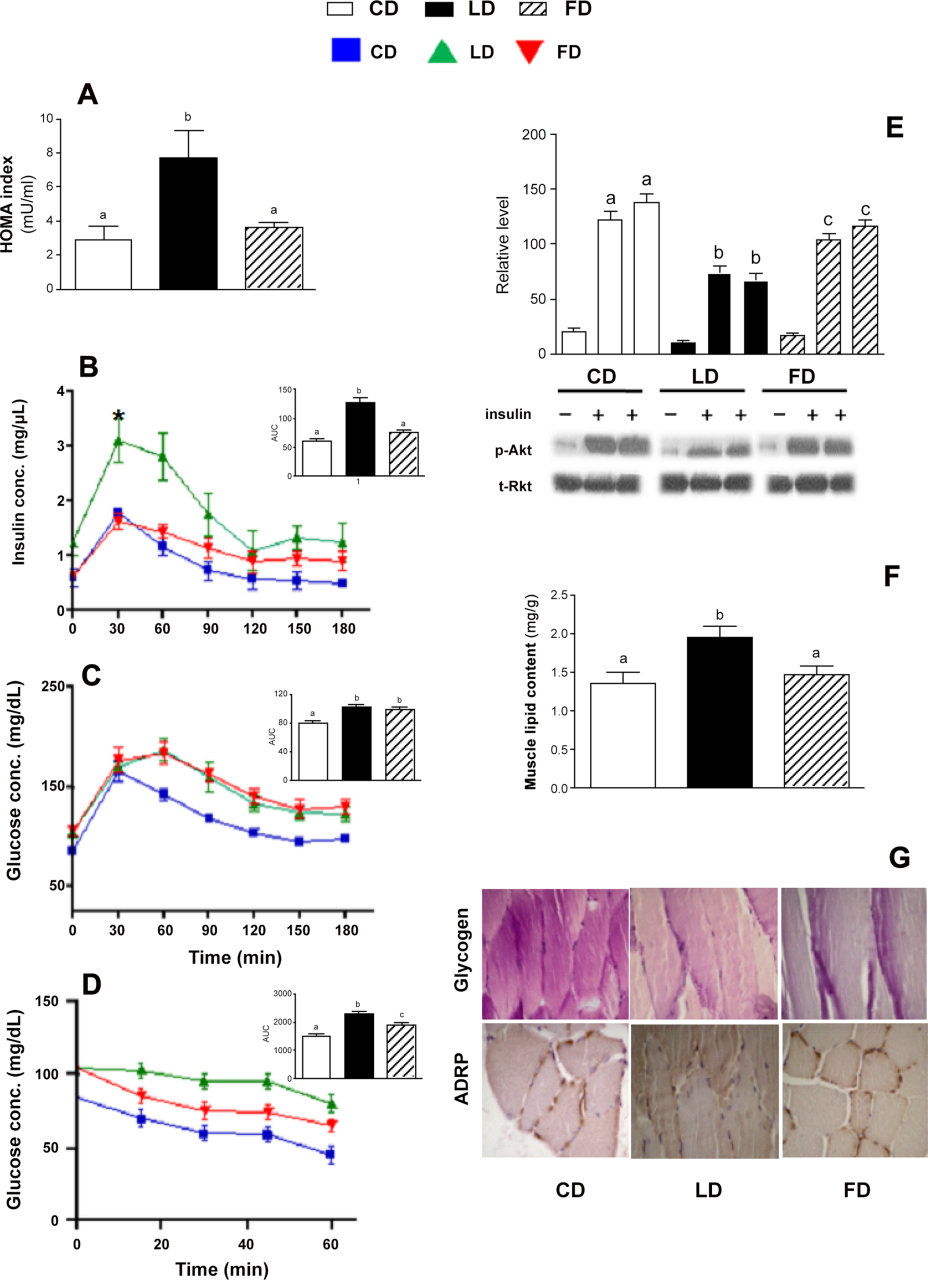
Effect of ω-3 PUFA on glucose and lipid metabolism. HOMA-IR index (A); Plasma insulin (B), and glucose (C) concentrations at different time intervals after glucose load and respective area under curve (AUC) (upper inserts) and insulin tolerance test (D) are shown. Representative western blots of insulin-induced Akt phosphorylation (Ser473) (E), and lipid content (F)in skeletal muscle are also reported. The graphic reported in panel 1E represent the densitometric analysis of protein band obtained in three separate experiments. Haematoxylin-eosin sections of glycogen (G upper panels), ADRP expression (G lower panels) are shown. PAS positive material was stained magenta at a magnification of 20x. Values are expressed as means±SEM from n = 8 animals/group. Different superscripted letters indicate statistically significant differences (P<0.05).

The HOMA-IR index was significantly greater in LD than in CD or FD-fed animals (Fig 1A). Accordingly, FD rats exhibited a higher tolerance to glucose loading than LD rats; in fact, despite similar glucose levels, FD rats exhibited a significantly lower insulin concentration and a reduced insulin area under the curve (AUC) than LD rats (Fig 1B and 1C),. Insulin tolerance test revealed less glucose reduction, following insulin administration, in LD and FD rats compared to control animals (Fig 1D), however rats on ω-3PUFA high fat diet showed an increased reduction of glucose levels compared to LD, indicating that fat present in the diet (lard or fish oil) can differently modify glucose metabolism. In fact, even if FD did not improve glicemic profile HOMA and insulin secretion results in part recovered, suggesting that in high fat diet the substitution of lard with ω-3PUFA can ameliorate insulin sensitivity.

Data obtained on insulin signal transduction on skeletal muscle support this hypothesis.

We evaluated Akt phosphorylation in skeletal muscle by western blot analysis after in vivo stimulation with the hormone. Insulin-stimulated Akt phosphorylation was less in LD-fed rats than in FD or CD-fed animals (Fig 1E). Significantly higher total lipid content in skeletal muscle (Fig 1F) and the widest glycogen-depleted areas in PAS sections were observed in LD-fed rats (Fig 1G upper panels). LD animals also exhibited a weaker immunostaining signal for ADRP in the muscle periphery and around adipocytes in connective tissue (Fig 1G lower panels), while it was similar in CD and FD.

### Mitochondrial function: oxidative capacity, efficiency, and oxidative stress

**Fig 2:**
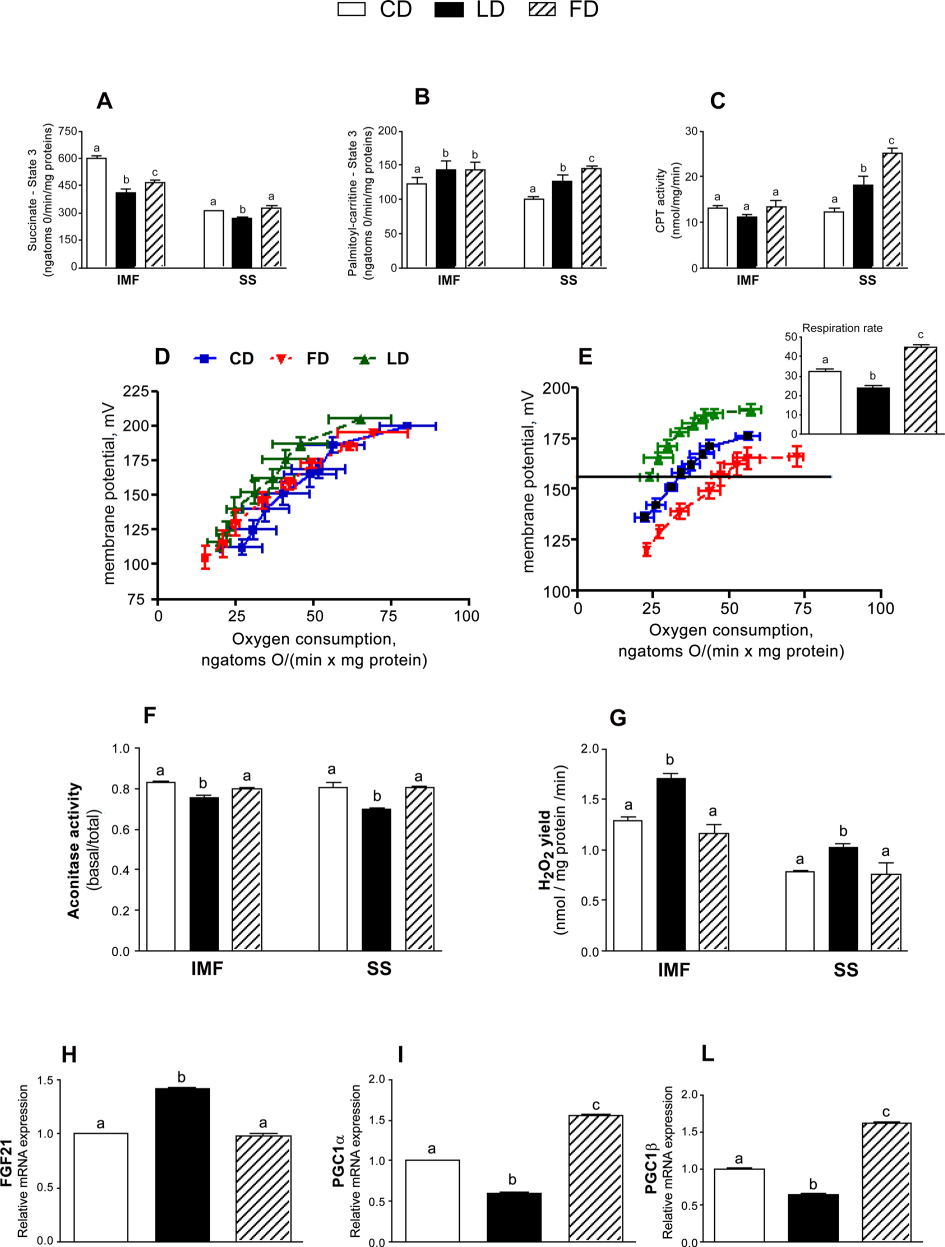
Effect of ω-3 PUFA on mitochondrial functions and energy efficiency. IMF and SS mitochondrial respiration in the presence of succinate (A) or palmitoyl-carnitine (B) as substrates were determined. CPT activity (C); basal (D) or palmitate-induced (E) proton leakage in SS mitochondria and respiration rates at 160 mV (the highest membrane potential common to all the curves) (upper insert); aconitase activity (F); H_2_O_2_ yield (G), relative mRNA expression of FGF21 (H), PGC1α (I), PGC1β (L) are also shown. Values are expressed as means±SEM from n = 8 animals/group. Different superscripted letters indicate statistically significant differences (P<0.05).

We found that IMF mitochondria from LD or FD-fed rats exhibited a significant lower State 3 respiration rate than control in presence of succinate, as substrate; conversely, State 3 in SS sub-population of LD-fed rats was less than that of FD- or CD-fed rats (Fig 2A). State 3, in presence of palmitoyl carnitine, was similar in IMF sub-population of LD and FD-fed animals and significantly higher than those measured in CD rats (Fig 2B). Conversely, in the same conditions, SS subpopulation from LD and FD showed an higher State 3 compared to CD-fed animals. Notably, SS mitochondrial sub-populations of FD exhibited a further increase in State 3 compared to LD group, paralleled by higher levels of CPT activity; no significant difference was found in CPT activity in the IMF sub-population (Fig 2C).

Proton-leakage from IMF was not influenced by the different diets (data not shown), and comparable basal proton-leak values were measured in SS mitochondria obtained from different groups (Fig 2D). With regard to fatty-acid-induced proton leak (measured using physiological amounts of palmitate), SS mitochondria of LD rats had the lowest proton leak among the three groups, and FD rats had the highest proton leak among the three groups analysed (Fig 2E). To compare the kinetic curves, oxygen consumption was also reported at a membrane potential of 160 mV (the highest membrane potential common to all obtained curves). FD-fed rats consumed more oxygen than LD- or CD-fed rats to maintain a given membrane potential (Fig 2E, upper insert). In IMF and SS mitochondrial populations, a significantly lower aconitase activity (Fig 2F) and a higher H_2_O_2_ yield (Fig 2G) clearly demonstrated that LD feeding enhanced the levels of pro-oxidant markers in muscle mitochondria compared to FD and CD regimens (P<0.01).

In addition, we analyzed the expression of the FGF21, PGC1α and PGC1β genes. FGF21 mRNA levels in LD rats were significantly increased compared with CD and FD rats (Fig 2H). PGC1-α and PGC1-β mRNA levels significantly increased in FD compared to the other groups and significantly decreased in LD compared to the other groups (Fig 2I–2L).

**Fig 3:**
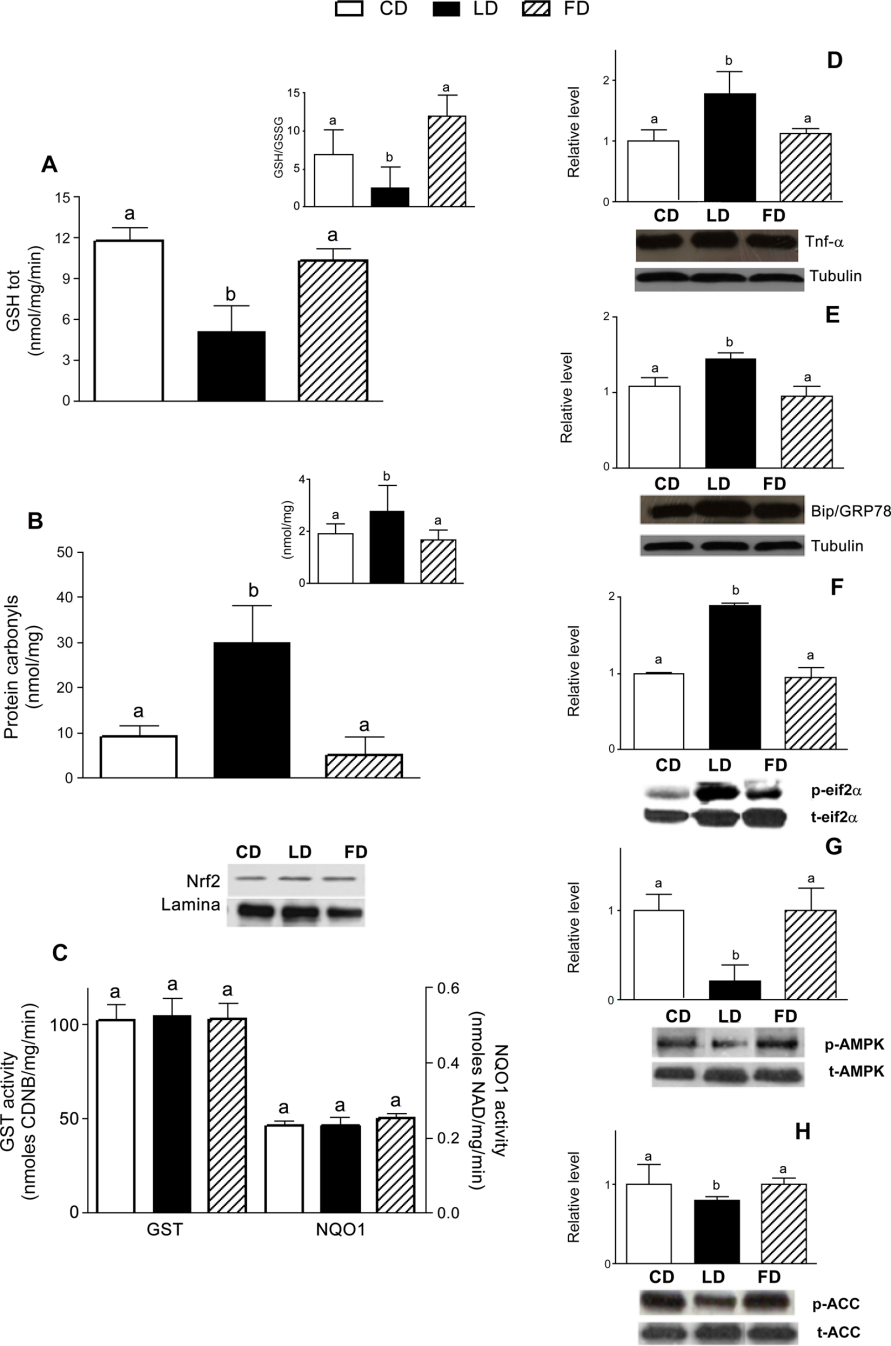
Effect of ω-3 PUFA on oxidative- and ER-stress and AMPK activation. Total thiols (A) and GSH/GSSG ratio (upper insert); protein carbonyl levels in skeletal muscle (B) and in serum (upper insert). Cytoplasmic GST and NQO1 activities and Nrf2 levels in nucleus (C). Representative immunoblots of TNFα (D) and BiP/GRP78 (E) p-eIF2-α (F), pAMPK (G) and pACC (H) are shown. Densitometric analysis of protein bands are reported: after normalisation the levels are expressed as the density ratio of target to control (tubulin, lamin or total protein). Values are expressed as means±SEM from n = 8 animals/group. Different superscripted letters indicate statistically significant differences (P<0.05).

The protective effects produced by FD feeding on skeletal muscle redox status were clearly indicated by the significant increased GSH/GSSG ratios respect to LD (Fig 3A upper insert). Moreover, the pro-oxidant effect produced by LD intake was demonstrated by a significantly lower GSH content (Fig 3A) and higher PC level in skeletal muscle (Fig 3B) and in serum (Fig 3B upper insert); these parameters were unchanged between FD rats and controls (Fig 3A and 3B), confirming the beneficial effect of fish oil in the high fat diet.

The negligible differences in Nrf2 protein translocation into cell nuclei together with comparable GST and NQO1 activities measured in skeletal muscle (Fig 3C) indicate that the Nrf2 pathway is not involved in both different high fat diet-mediated modulation of redox status.

Increased TNFα, BiP/GRP78 and eIF2-α levels (Fig 3D–3F) were detected in the skeletal muscle of LD, whereas the levels in FD rats were similar to CD rats. Finally, pAMPK protein content was significantly lower in the skeletal muscle of LD-fed rats than in those of the other experimental groups (Fig 3G). LD-diet exhibited an inhibitory effect on AMPK activity, as indicated by a concomitant decline of ACC phosphorylation (Fig 3H), which is an indicator of AMPK activity [41].

## Discussion

The main finding of this study is that the intake of ω-3 PUFAs enriched diet, at high percentage, reduces fat accumulation in skeletal muscle and decreases metabolic/mitochondrial efficiency, attenuating insulin resistance, ER- and oxidative-stress, compared to an isocaloric high fat diet rich in SFAs. Moreover, SS mitochondria were identified as the main target of diet-induced alterations in function and efficiency. The effects exerted by ω-3 PUFAs intake have been associated to mitochondrial uncoupling and AMPK/ACC, rather than to Nrf2 pathway activation in skeletal muscle.

Here, LD-feeding was associated with high metabolic efficiency, weight gain, body lipid levels, and also with metabolic alterations, such as dyslipidemia, and insulin resistance, accompanied by an increase in low grade inflammation compared to a standard diet. As reported, despite comparable ME intake between LD and FD groups, the substitution in lard-based diet with ω-3 PUFA in high fat fed animals showed a reduction in metabolic efficiency, body weight and body lipid levels, accordingly with a correction of dyslipidemia and insulin resistance. These data are consistent with previous findings, indicating a reversal of insulin resistance by ω-3 PUFA intake [42,43]. The effects on body weight and lipids, we observed in FD-fed rats, can be explained, at least in part, by an increase in energy expenditure/O_2_ consumption and reduced energy efficiency. Moreover, the decreased RQ index observed in LD and FD-fed animals, which reflects the ratio of carbohydrate to fatty acid oxidation, demonstrates that these animals used a higher amount of fatty acids, as a fuel source, compared to controls. These data indicate that FD intake improves the ability to utilise fat, as a metabolic fuel, which suggests that the large part of the higher energy intake was dissipated through increased metabolic activity in these animals.

An interesting finding is that high-fish oil diet despite to any effect on glycaemia in confront of LD, it is able to attenuate the development of insulin resistance, preventing the alteration of glucose tolerance related to an impairment of insulin signalling due to fat over nutrition. The improvement in insulin sensitivity may be, at least in part, a consequence of the anti-inflammatory effect of ω-3 PUFAs in this nutritional model. Our previous study showed that high-fish oil diet attenuated the development of systemic and tissue inflammation [23] and decreased hepatic lipid accumulation through improved mitochondrial fatty acid utilization supported by mitochondrial uncoupling [24].

Here, HOMA index and oral glucose and insulin tolerance tests showed that high-fish oil diet attenuated alteration of glucose homeostasis compared to an isocaloric lard-based diet, indicating that fat present in the diet (lard or fish oil) can differently modify insulin sensitivity.

Consistently, at skeletal muscle level, FD also attenuated tissue insulin resistance, modulating insulin signalling, restoring protein kinase B (PKB/Akt) phosphorylation and decreased lipid accumulation, increasing ADRP levels, involved in the proper TG storage [44], and AMPK activation. All these metabolic effects of fish oil fat diet are strengthened by the suppression of inflammatory process, evidenced by reduced serum levels of TNFα, MCP-1 and LPS, and of ER stress at skeletal muscle level, where GRP78 expression and eif2α activation were down-regulated.

The effects of ω-3 PUFA overload on the prevention of weight gain excess and the development of insulin resistance may be mediated by adiponectin and leptin, two adipokines that regulate glucose and lipid metabolism, through AMPK activation [11,12]. Our results demonstrate decreased serum leptin levels in FD, consistently with fat mass reduction, compared to LD rats, and restored serum adiponectin levels to those of CD rats, suggesting a key role of fish oil in the reduction of the development of insulin resistance in an animal model of fat overnutrition. The reduced adiponectin values found in LD rats were consistent with those reported by Kalupahana et al. [45]. It is well known the lipid sensor activity of AMPK, an important metabolic regulator [46]. Notably, the activation of AMPK exhibits multiple protective effects, including a reduction in inflammation, oxidative stress and insulin resistance [46]. Recently it has been reported that AMPK activation protects against lipid-induced disorders [6,7] by reducing ER stress. Previous findings by Jelenik et al. [22] showed that ω-3 PUFAs intake induced AMPK activation in liver, reverting insulin resistance and steatosis in mice.

Here, we found that FD, in a different way by LD, modulated AMPK/ACC pathway restoring adiponectin and fatty acid metabolism in skeletal muscle. Skeletal muscle is the primary tissue involved in the regulation of glucose metabolism, energy expenditure and lipid utilization and it is inherently linked to the development of whole-body insulin resistance [47]. The recognized link between insulin resistance and mitochondrial dysfunction [3,48] prompted us to evaluate the effect of dietary fat regimens on mitochondrial oxidative capacity, energy efficiency, and oxidative stress in both SS and IMF mitochondrial populations. Indeed, SS mitochondria provide energy for membrane-related processes, such as substrate oxidation and insulin action, whereas IMF mitochondria support muscle contraction [49]. In addition, SS mitochondria may be more susceptible to damage by ectopic lipid deposition, because lipid content decreases exponentially from immediately below the sarcolemma toward the central region of the muscle fibre [50]. Because of their proximity to the sarcolemmal membrane, SS mitochondria can more easily interfere with key proteins involved in the insulin-signalling cascade [50], anyway a pivotal role in insulin-mediated glucose transporter trafficking has been shown also for t-tubules structure [51]. Interestingly, we demonstrate that both fat diets mainly affects SS, rather than IMF mitochondria, indicating that the observed mitochondrial alterations may contribute to the pathogenesis of insulin resistance. In particular, no difference was observed in IMF mitochondria uncoupling, while SS subpopulation demonstrated an increased uncoupling in FD fed rats.

Association between diet-induced ectopic fat storage in skeletal muscle and mitochondrial dysfunction is well known [3,48]. Accordingly, LD-rat skeletal muscle mitochondria exhibited reduced respiratory capacity, as indicated by the decrease in succinate State 3 oxygen consumption, and increased oxidative stress, even when the ability to utilize fat as a metabolic fuel was elevated. The increased mitochondrial fatty acid oxidation could be a result of an diet-induced increase of FFA uptake and/or enhanced CPT activity which would further increase the entry of long-chain FFA into the mitochondria. However, as such increased lipid oxidation is likely not sufficient to handle the greater FFAs load, resulting in the ectopic triglyceride storage in skeletal muscle. Moreover, a further mechanism contributing to fat accumulation can be the increase in mitochondrial efficiency, as shown by the decrease in the induced proton leak in LD rats. Therefore, a higher mitochondrial efficiency, suggestive of a reduced amount of substrate to be burned to obtain ATP, together with an increase in NEFA serum levels could account for the triglyceride accumulation in skeletal muscle.

In our experimental conditions, mitochondrial dysfunction in LD rats was related to an increase in FGF21 gene expression in skeletal muscle, which follows a stress response [52]. Increased mitochondrial oxidative stress parameters were found in LD rats as showed by hydrogen peroxide yield, aconitase activity, protein carbonyls amount and GSH/GSSG ratio. This effect can be attributable to the concomitant increase in fatty acid oxidation rate, resulting in NADH and FADH_2_ generation and thus electron delivery to the respiratory chain, and to respiratory chain impairment (as indicated by the decrease in succinate State 3 oxygen consumption, which would partially block electron flow within the respiratory chain). Further, the decreased proton leak can contribute to excessive ROS formation [53] in LD rats. In fact, one of the postulated roles of uncoupling is known to be the maintenance of mitochondrial membrane potential below the critical threshold for ROS production [54].

The improvement of respiratory capacity, fatty acid oxidation, CPT activity and the decreased mitochondrial efficiency, exhibited by the FD rats, may be interpreted as the result of converging protective mechanisms against insulin resistance. In addition, the significant alteration of all the considered oxidative stress markers mirrors the differential ability of ω-3 PUFA- and SFA-based diets to trigger oxidative stress in skeletal muscle [43]. In addition, in FD-fed animals the increased respiratory capacity is associated with the increase in gene expression levels of FGF-21, PGC-1α and PGC-1β, involved not only in the regulation of mitochondrial activities and biogenesis [55–58], but also to the development of insulin resistance in skeletal muscle [59]. The increase in mitochondrial biogenesis was supported by changes in mitochondrial protein mass calculated by two different approaches, namely 1) by measuring the activity of a mitochondrial marker enzyme citrate synthase in skeletal muscle homogenates and in isolated SS and IMF mitochondria and 2) by evaluating the mitochondrial yield (i.e., milligrams isolated protein per gram starting wet tissue) in each mitochondrial subpopulation. Independent of the methodology applied, we found that the mitochondrial mass was significantly increased in both SS and IMF compartments in FD rats and significantly reduced in LD rats (see data in S1 Text). Therefore, ω-3 PUFAs, in addition to their positive effect on mitochondrial respiration, also act on the level of mitochondrial gene expression in skeletal muscle cells: the increase in PGC-1α should play a role in the recovery of mitochondrial respiration, promoting both oxidative phosphorylation-linked and uncoupling-linked respiration in differentiated myotubes oxidative capacity in skeletal muscle cells [60–62].

Conversely, the negligible alteration of Nrf2-pathway, which plays a key role in cellular protection against oxidative stress, is consistent with evidence demonstrating that transient and low-levels of ROS are needed for Nrf2-antioxidant responsive element pathway activation [63].

In conclusion, our data strengthened the capability of high dietary PUFA intake to reduce fat mass and insulin resistance associated to fat overnutrition, modulating energy efficiency. In particular, at skeletal muscle level, ω-3 PUFAs enriched diet promotes inefficient metabolism, generating heat instead of ATP, increases lipid oxidation, activating the pathway AMPK/ACC, and reduces ROS generation in mitochondria. Therefore, modulating mitochondrial function and efficiency in the skeletal muscle, they lessen pro-inflammatory, pro-oxidant signs and insulin resistance also in condition of nutritionally-induced obesity.

## Supporting Information

S1 Text Detailed analysis for mitochondrial protein mass evaluation. (DOC)

## Abbreviations

ER: Endoplasmic reticulum
AMPK: AMP-activated protein kinase
ROS: reactive oxygen species
PUFA: polyunsaturated fatty acids
DHA: docosahexaenoic acid
EPA: eicosapentaenoic acid
SFA: saturated fatty acids
CD: control diet
FD: high fat diet containing ω-3 PUFA
LD: high; fat diet containing lard
SS: subsarcolemmal mitochondrial subpopulation
IMF: intermyofibrillar mitochondrial subpopulation
ME: metabolizable energy
HOMA: homoeostatic model assessment
NEFAs: non-esterified fatty acids
LPS: lipopolysaccharide
MCP: monocyte chemoattractant protein
CPT: carnitine palmitoyl transferase
ADRP: adipocyte differentiation-related protein
NQO: NADPH quinone oxidoreductase
GRP: glucose regulated protein
eIF: eukaryotic translation initiation factor
PGC: peroxisome proliferator-activated receptor γ coactivator
FGF: fibroblast growth factor
