# Supplementary material for: Polyunsaturated Fatty Acids Attenuate Diet Induced Obesity and Insulin Resistance, Modulating Mitochondrial Respiratory Uncoupling in Rat Skeletal Muscle
Source: PLoS One. 2016 Feb 22;11(2):e0149033. doi: 10.1371/journal.pone.0149033 (PMC4762694; doi:10.1371/journal.pone.0149033)
Supplement: S1 Text — (DOC) [file pone.0149033.s001.doc]

**S1 appendix**

**Materials and methods**

Mitochondrial protein mass was assessed by measuring the activity of citrate synthase (CS) in skeletal muscle homogenates and in isolated mitochondria, according to Srere (Srere PA. Citrate synthase. Meth Enzymol 1969; 13:3–5), and also by evaluating the mitochondrial yield. In the first approach, CS activity, measured in the homogenate and expressed per gram wet muscle, reflects the product of mitochondrial protein mass and specific activity of the CS enzyme. To determine CS specific activity, measurements were made in isolated SS and IMF mitochondria, and the results were expressed per milligram mitochondrial protein. In addition, to determine protein mass in each mitochondrial subpopulation, CS activity in IMF and SS mitochondria was expressed per gram (wet tissue). Purity of IMF and SS mitochondrial preparations was determined by the degree of contamination by other ATPase-containing membranes (<10%). In the second approach, mitochondrial SS and IMF protein mass was evaluated as mitochondrial protein yield and the milligram of mitochondrial proteins (obtained from both mitochondrial subpopulations) was expressed per gram wet tissue. Indeed, changes in mitochondrial yield could result from 1) changes in the amount of mitochondria in the starting tissue or 2) changes in the sedimentation characteristics of the organelles. To exclude the possibility that changes in mitochondrial yield could result from loss of mitochondria during the isolation procedure, the recovery of CS activity in the various fractions was assessed during the isolation procedure of SS and IMF mitochondria.

**Results**

Mitochondrial SS and IMF protein mass from skeletal muscle at the end of treatment was determined by using the mitochondrial marker enzyme CS. After 6 weeks of treatment, the specific activity of CS per gram tissue was found to be significantly lower in LD rats compared to controls and FD rats, both in muscle homogenate and in isolated SS and IMF mitochondria, whereas the same parameters were significantly increased in FD rats compared to the other two groups. The CS specific activity per milligram protein in SS and IMF mitochondria was found to be significantly lower in LD rats compared to controls and FD rats (S 1 Table).

Therefore a significantly lower mitochondrial protein content (calculated as the ratio between citrate synthase activity in the homogenate and isolated mitochondria) was observed in LD rats and significantly higher mitochondrial protein content was observed in FD-treated rats indicating that the improved oxidative capacity appears to be supported (at least in part) by an increased mitochondrial mass (S 1 Table).

In addition, the yield of both SS and IMF mitochondria from LD rats was lower than that from FD and control rats and increased in FD rats than other two groups (S1 Table), whereas recovery of citrate synthase activity was unchanged in the various fractions excluding the possibility that changes in mitochondrial yield could result from loss of mitochondria during the isolation procedure (S1 Figure).

# **S1 Table**

|  | **CD** | **LD** | **FD** |
| --- | --- | --- | --- |
| CS activity (μmol/min x g wet tissue) |  |  |  |
| Homogenate | 18.9±1.1^a^ | 13.5±0,9 ^b^ | 25.3±1.0 ^c^ |
| IMF | 6.2±0.4^a^ | 4.3±0.1 ^b^ | 8.1±0.2 ^c^ |
| SS | 2.2±0.05^a^ | 1.5±0.1 ^b^ | 3.1±0.2 ^c^ |
| CS activity (μmol/min x mg protein) |  |  |  |
| IMF | 3.15±0,1 ^a^ | 2.64±0,09 ^b^ | 2.91±0.1 ^a^ |
| SS | 3.9±0,09 ^a^ | 3.4±0,1 ^b^ | 4.12±0,1 ^a^ |
| Protein yield (mg/g tissue) |  |  |  |
| IMF | 1.98±0.02^a^ | 1.60±0.01 ^b^ | 2.80±0.16 ^c^ |
| SS | 0.57±0.03 ^a^ | 0.46±0.01 ^b^ | 0.74±0.03 ^c^ |

**S1 Figure**

**Figure 1S. Percent recovery of citrate synthase activity during mitochondrial isolation procedure. Values are the means ± SE from n=8 animals/group. supSS, SS supernatant; supIMF, IMF supernatant.**
